# Inhibition of Uterine Contractility by Thalidomide Analogs via Phosphodiesterase-4 Inhibition and Calcium Entry Blockade

**DOI:** 10.3390/molecules21101332

**Published:** 2016-10-07

**Authors:** Eduardo Fernández-Martínez, Héctor Ponce-Monter, Luis E. Soria-Jasso, Mario I. Ortiz, José-Antonio Arias-Montaño, Guillermo Barragán-Ramírez, Cynthia Mayén-García

**Affiliations:** 1 Centro de Investigación en Biología de la Reproducción, Área Académica de Medicina del Instituto de Ciencias de la Salud, Universidad Autónoma del Estado de Hidalgo, Pachuca 42090, Hidalgo, Mexico; poncemonterh@gmail.com (H.P.-M.); soriajasso@gmail.com (L.E.S.-J.); mario_i_ortiz@hotmail.com (M.I.O.); mayen_09@hotmail.com (C.M.-G.); 2 Departamento de Fisiología, Biofísica y Neurociencias, Centro de Investigación y de Estudios Avanzados del IPN, Apdo. Postal 14-740, México City 07360, Mexico; jaarias@fisio.cinvestav.mx; 3 Hospital General de los SSH, Pachuca 42070, Hidalgo, Mexico; gmobarragan@hotmail.com

**Keywords:** calcium blockers, cAMP, human myometrium, phosphodiesterase-4, preterm labor, relaxation, tocolytics, thalidomide analogs

## Abstract

Uterine relaxation is crucial during preterm labor. Phosphodiesterase-4 (PDE-4) inhibitors have been proposed as tocolytics. Some thalidomide analogs are PDE-4 inhibitors. The aim of this study was to assess the uterus-relaxant properties of two thalidomide analogs, methyl 3-(4-nitrophthalimido)-3-(3,4-dimethoxyphenyl)-propanoate (4NO2PDPMe) and methyl 3-(4-aminophthalimido)-3-(3,4-dimethoxyphenyl)-propanoate (4APDPMe) and were compared to rolipram in functional studies of spontaneous phasic, K^+^-induced tonic, and Ca^2+^-induced contractions in isolated pregnant human myometrial tissues. The accumulation of cAMP was quantified in HeLa cells. The presence of PDE-4B2 and phosphorylated myosin light-chain (pMLC), in addition to the effect of thalidomide analogs on oxytocin-induced pMLC, were assessed in human uterine myometrial cells (UtSMCs). Thalidomide analogs had concentration-dependent inhibitory effects on spontaneous and tonic contractions and inhibited Ca^2+^-induced responses. Tonic contraction was equipotently inhibited by 4APDPMe and rolipram (IC_50_ = 125 ± 13.72 and 98.45 ± 8.86 µM, respectively). Rolipram and the thalidomide analogs inhibited spontaneous and tonic contractions equieffectively. Both analogs increased cAMP accumulation in a concentration-dependent manner (*p* < 0.05) and induced changes in the subcellular localization of oxytocin-induced pMLC in UtSMCs. The inhibitory effects of thalidomide analogs on the contractions of pregnant human myometrium tissue may be due to their PDE-4 inhibitory effect and novel mechanism as calcium-channel blockers.

## 1. Introduction

Preterm (premature) labor is the major cause of neonatal morbidity and mortality, with a worldwide average rate of 12% that includes low and high income countries and accounts for 75%–85% of all neonatal deaths [1,2,3,4]. Preterm labor is defined as delivery occurring before 37 completed weeks of gestation [3], and its physiology is complex and not yet fully understood [4]. Causes of preterm labor are drug abuse, multiple pregnancies, previous preterm delivery, asymptomatic bacteriuria and bacterial vaginosis, among others [3,4]. There is strong evidence that infection and/or inflammation may be a primary causal mechanism (up to 25%) of preterm birth [3,4,5,6].

The uterus is considered a myogenic organ because it contracts spontaneously due to waves of electrical activity that provoke membrane depolarization and result in a rise in [Ca^2+^]_i_. The uterus also responds to many agonists; thus, changes in the expression of receptors and the activation of signaling pathways in myocytes may be involved in the regulation of uterine contractility [1,7]. The uterine quiescence maintained during pregnancy and the increased activity during the spontaneous onset of parturition may be associated with functional changes in myometrial receptors [8]. Uterine infections induce the production and release of proinflammatory cytokines, such as tumor necrosis factor (TNF)-α, interleukin (IL)-6 and IL-1β, which, in turn, activate phospholipid metabolism, arachidonic acid release and the signal transduction system involving nuclear factor (NF)-κB to increase the expression of cyclooxygenase (COX)-2, which provokes the production of prostaglandin (PG)-E2 and PG-F2α, both of which stimulate contractility by activating the phospholipase-C/Ca^2+^ (PLC) pathway. Cytokines and eicosanoids interact to accelerate normal parturition, resulting in preterm labor [1,3,4,5,6,8,9]. However, the second messenger adenosine 3′,5′-cyclic monophosphate (cAMP) inhibits cytokine production and promotes the relaxation of the myometrium by activating cAMP-dependent protein kinase (PKA), which phosphorylates the myosin light–chain (MLC) kinase (MLCK), decreasing the affinity of this enzyme for the Ca^2+^–calmodulin complex. PKA may promote smooth muscle relaxation by inhibiting PLC activation and Ca^2+^ entry mechanisms and by activating Ca^2+^–stimulated K^+^ channels and Ca^2+^ pumps [7,10,11]. Intracellular cAMP levels result from the interaction of a wide range of agonists with G protein-coupled receptors (GPCRs), which ultimately activate (via Gαs) or inhibit (via Gαi) adenylyl cyclase (AC), the enzyme family responsible for generating cAMP from adenosine-5′-triphosphate (ATP). Agonists that elevate intracellular cAMP induce myometrial relaxation [10,11]. In contrast, phosphodiesterase (PDE)-4 catalyzes the hydrolysis and inactivation of cAMP. The major source of PDE activity in human myometrium is represented by PDE-4 isoforms. Moreover, the contribution of the PDE-4 family increases in late pregnancy, reaching 75% of the total cAMP-PDE activity. Consequently, these enzymes are a pharmacological target [7,12,13].

The use of tocolytic drugs to inhibit uterine contractility is controversial, including those that operate through cAMP elevation, such as β-adrenoceptor agonists, because there is no evidence that the currently available drugs improve long-term neonatal outcomes. Some tocolytics can cause serious cardiovascular, metabolic and neuromuscular side effects [1,3,4,11]. Thus, PDE-4 inhibitors appear to be promising drugs for the treatment of preterm labor by increasing the levels of cAMP [7,12,13,14]. Indeed, several PDE-4 inhibitors are currently being evaluated in clinical trials for diverse inflammatory diseases [15,16]. In this regard, thalidomide and its analogs have emerged as immunomodulatory drugs for the treatment of diverse chronic inflammatory diseases [17,18,19]. Additionally, some novel thalidomide analogs are potent PDE-4 inhibitors without teratogenic effects, and they are more effective as anti-inflammatory agents [20,21,22,23,24]; however, their probable relaxant effects on smooth muscle contractions have not yet been reported. In preliminary experiments, two thalidomide analogs, 4NO2PDPMe and 4APDPMe, exhibited better inhibitory activity of uterine muscle contractions in murine species in comparison to some other analogs. The aim of this study was to assess the uterus-relaxant properties in pregnant human myometrium of the two thalidomide analogs, 4NO2PDPMe and 4APDPMe, as PDE-4 inhibitors and potential Ca^2+^-channel blockers.

## 2. Results

### 2.1. Thalidomide Analogs and Rolipram Inhibit Spontaneous and Tonic Contractions

**Figure 1:**
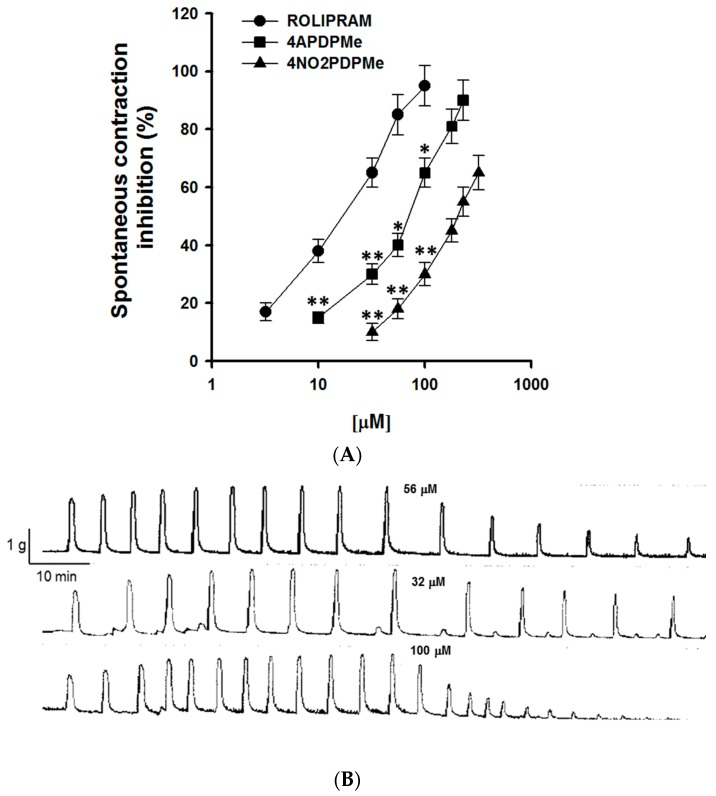
Inhibitory effects of rolipram and thalidomide analogs on the spontaneous contraction of pregnant human myometrium. (**A**) Concentration-effect curves of rolipram, 4NO2PDPMe and 4APDPMe on the spontaneous contractions of pregnant human uterine strip preparations; each point represents the mean of 6 experiments, and vertical bars indicate the standard error of the mean (±SEM); (**B**) Typical recording of spontaneous phasic contraction inhibition by a thalidomide analog in a concentration-dependent manner. Difference vs. rolipram, * *p* < 0.05 or ** *p* < 0.001.

Myometrial smooth muscle is myogenic and can generate contractions spontaneously without the need for external stimulation [25]. Figure 1A shows the concentration-dependent sigmoid curves of the inhibitory effect on the spontaneous phasic contraction of pregnant uterine smooth muscle by thalidomide analogs and rolipram. Rolipram was the most potent drug, followed by 4APDPMe, which was more potent than 4NO2PDPMe; however, all of the drugs were equally effective, as described below. Figure 1B shows a typical tracing of the inhibition of spontaneous phasic contractions by thalidomide analog addition in a concentration-dependent manner.

**Figure 2:**
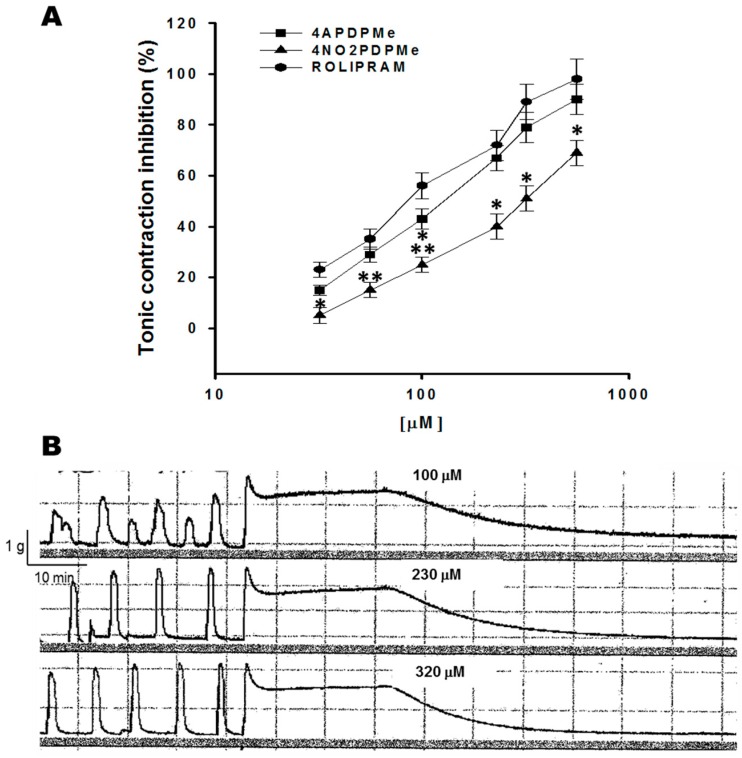
Inhibitory effects of rolipram and thalidomide analogs on the tonic contraction of pregnant human myometrium. (**A**) Concentration-effect curves of rolipram, 4NO2PDPMe and 4APDPMe, on 40 mM KCl–induced tonic contractions of pregnant human uterine strip preparations; each point represents the mean of 6 experiments, and vertical bars indicate the standard error of the mean (±SEM); (**B**) Typical recording of tonic contractions inhibited by a thalidomide analog in a concentration-dependent manner. Difference vs. rolipram, * *p* < 0.05 or ** *p* < 0.001.

Tonic contraction of smooth muscle was induced by a depolarizing KCl solution that stimulates voltage-gated calcium channels [26,27]. Figure 2A shows concentration–dependent inhibitory effects of thalidomide analogs and rolipram on tonic contraction generated by the depolarization of high K^+^. Rolipram and 4APDPMe were equipotent as inhibitory agents (*p* < 0.05), whereas 4NO2PDPMe displayed a very distinct concentration–response curve compared with the other agents. However, all of them were equally effective, as described below. Figure 2B shows a typical tracing of the concentration-dependent relaxant effects of a thalidomide analog on the tonic contraction of pregnant human myometrium.

A summary of the IC_50_ and Emax values for both thalidomide analogs and rolipram are presented in Table 1, all of which were derived from the concentration–response curve analysis. Spontaneous contractions of the myometrium appeared to be more sensitive to the inhibitory effects of the three compounds when compared with tonic contractions because their IC_50_ values were lower than the IC_50_ required during K^+^-induced sustained contractions. Rolipram was the most potent inhibitor of spontaneous contractions, although it and 4APDPMe had equipotent effects on tonic contractions, and 4NO2PDPMe presented the highest IC_50_ values for both myometrial contractions (*p* < 0.05). Furthermore, comparisons of Emax showed that rolipram and thalidomide analogs were statistically equally effective for both contractions.

### 2.2. Calcium Entry Blockade as a Possible Uterus–Relaxant Mechanism of Thalidomide Analogs and Rolipram

**Figure 3:**
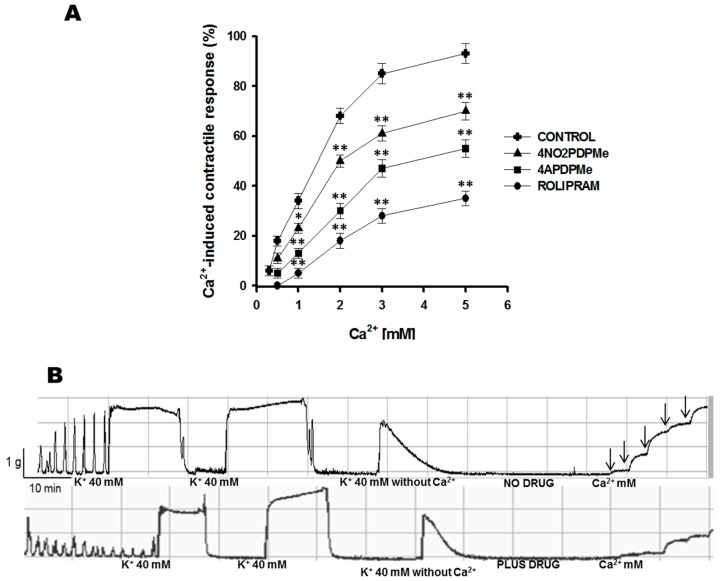
Inhibitory effects of rolipram and thalidomide analogs on Ca^2+^-induced contractions of pregnant human myometrium. (**A**) Concentration-effect curves of the Ca^2+^-induced tonic contractile response (40 mM KCl) of pregnant human uterine strip preparations in the presence of the respective IC_50_ concentrations of rolipram (98.45 µM), 4APDPMe (125 µM), and 4NO2PDPMe (203.45 µM); each point represents the mean of 6 experiments, and vertical bars indicate the standard error of the mean (±SEM); (**B**) Typical recording of tonic contractions induced by cumulative Ca^2+^ concentrations and inhibited by a thalidomide analog. Difference vs. rolipram, * *p* < 0.05 or ** *p* < 0.001.

Both analogs showed fast uterus–relaxant activity toward either spontaneous or tonic contractions; thus, based on the disappearance of the compounds within a short time after their addition, they had a rapid inhibitory effect on the amplitude and/or frequency of the contractions. These results strongly suggested an alternative cell membrane–mediated effect, such as calcium channel blockade, in addition to cytoplasmic PDE-4 inhibition; thus, an experiment was conducted to explore possible mechanisms of action. The development of K^+^-induced tension in isolated uterine smooth muscle was reduced by lowering the Ca^2+^ concentration in the bathing medium [28]. In this respect, an almost complete recovery of the myometrial contractile response was achieved by the addition of cumulative Ca^2+^ concentrations to the bath of isolated uterine strips (Figure 3A), whereas prior incubation with the respective IC_50_ of thalidomide analogs or rolipram prevented this recovery of tonic contraction. Figure 3B shows a representative tracing of tonic contractions provoked by high K^+^ in medium containing Ca^2+^. Conversely, the contractions became transitory and were reduced in medium lacking Ca^2+^, but the contractile response recovered following the addition of calcium. However, the contractile response remained inhibited in uterine strips exposed to thalidomide analogs or rolipram. Furthermore, even in the presence of 5 mM Ca^2+^, the tissue was unable to recover 100% contraction in the presence of the compounds.

To date, this is the first report to demonstrate calcium entry blockade induced by rolipram or thalidomide analogs as a possible uterus–relaxant mechanism of action. Table 2 summarizes the comparative analysis of the drug activity effects on Ca^2+^-induced contractile responses, such as Ca^2+^ entry inhibitors. The efficacy was as follows: rolipram was the most effective (*p* < 0.05); rolipram > 4APDPMe > 4NO2PDPMe.

### 2.3. Thalidomide Analogs Increase cAMP Levels in HeLa Cells

**Figure 4:**
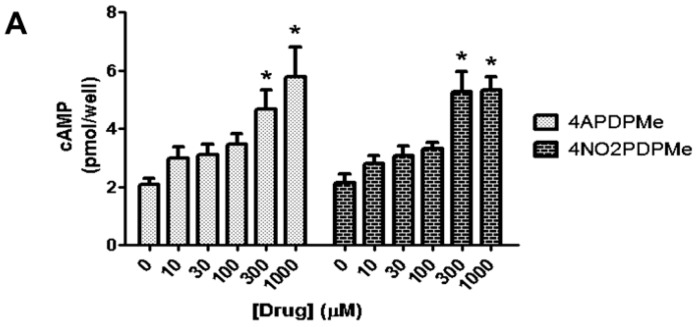

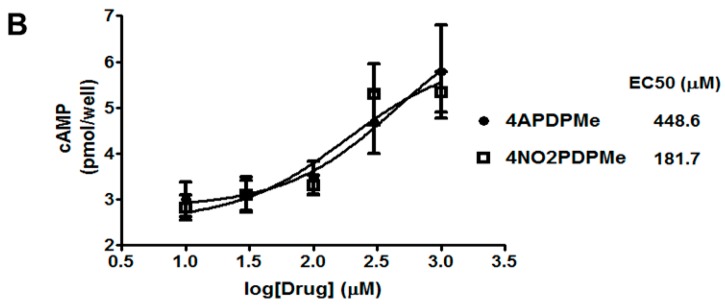
Intracellular cAMP levels induced by thalidomide analogs in HeLa cells. (**A**) Intracellular cAMP levels in HeLa cells in the presence of forskolin (3 µM) and 4APDPMe or NO2PDPMe (10–1000 µM); the analogs provoked cAMP accumulation in a concentration–dependent manner. Each bar represents the mean of 6 experiments ± standard error of the mean (SEM); (**B**) Concentration-effect curves of 4APDPMe and 4NO2PDPMe; their respective IC_50_ was calculated by a curve analysis. Each point represents the mean of 6 experiments; vertical bars indicate the standard error of the mean (±SEM). * Significantly different from basal levels without drug (*p* < 0.05), as determined by ANOVA followed by Bonferroni’s test.

Inhibition of PDE-4 augments intracellular levels of cAMP and, as a consequence, activates relaxant and immunomodulatory effects [13]. Accordingly, it was mandatory to demonstrate whether thalidomide analogs were capable of increasing intracellular cAMP levels; thus, uterine epithelial HeLa cells were selected as a well-known model in which other PDE inhibitors have been utilized [29,30]. Figure 4A shows the concentration-dependent increase in endogenous cAMP in HeLa cells induced by both thalidomide analogs, which reached marked augmenting effects at 300 and 1000 µM (*p* < 0.05). A concentration–response curve analysis was generated (Figure 4B) and, at least in this model, 4NO2PDPMe was significantly more potent than 4APDPMe as a cAMP–elevating agent (EC_50_ of 181.7 and 448.6 µM, respectively), despite the superior biological activity of 4APDPMe in all previous experiments. Additionally, 4APDPMe was three-fold more potent than IBMX as a cAMP-elevating agent in preliminary experiments conducted in CHO-K1 cells (data not shown).

### 2.4. PDE-4B2 and pMLC Expression in UtSMC, and the Effects of Thalidomide Analogs on OT–Induced pMLC Production

**Figure 5:**
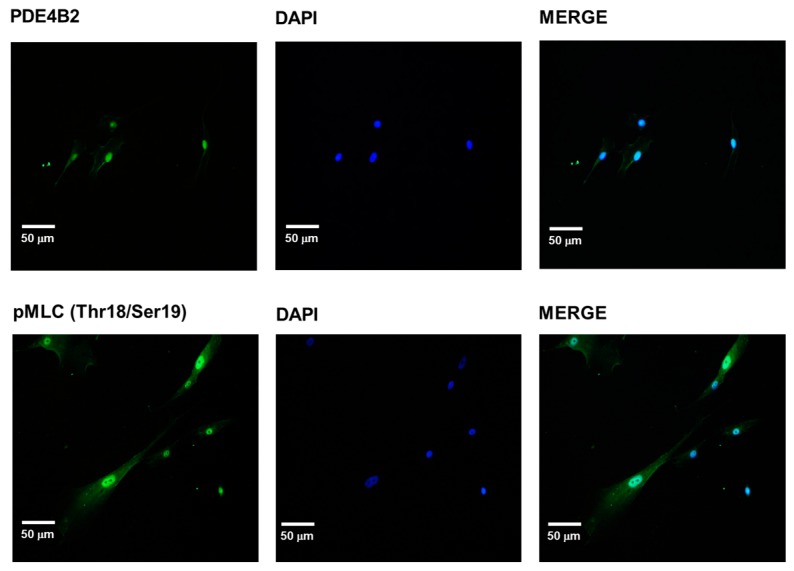
Expression of PDE-4B2 and pMLC in UtSMC cells. Normal subcellular localization of PDE-4B2 and pMLC was detected by immunocytochemistry in UtSMC cells; PDE-4B2 exhibited a perinuclear and cytosolic distribution, whereas pMLC was localized a perinuclear as well as cytosolic/cytoskeletal localization. DAPI: 4′,6-diamidino-2-phenylindole was used to stain the nucleus (blue).

The PDE-4 family includes multiple isoforms encoded by four genes (PDE-4A-D). The levels of the PDE-4D and PDE-4B genes are increased in pregnant women who are close to term. The isoform PDE-4B2 is involved in the regulation of the inflammatory response and is overexpressed at the end of pregnancy, with a concomitant change in the myorelaxant properties of PDE-4 inhibitors [12,31]. Increased cAMP levels in response to PDE-4 inhibition may provoke a reduction in the amount of pMLC, which induces relaxation because of the diminished affinity of MLCK for its substrate. Hence, it was important to perform an initial assessment of the presence of PDE-4B2 and pMLC in the cultured UtSMC cells to evaluate the effects of both thalidomide analogs on those proteins either alone or during OT stimulation. Figure 5 shows the quiescent perinuclear and cytosolic expression of PDE-4B2 and pMLC, which was not influenced by pregnancy in the present analysis.

**Figure 6:**
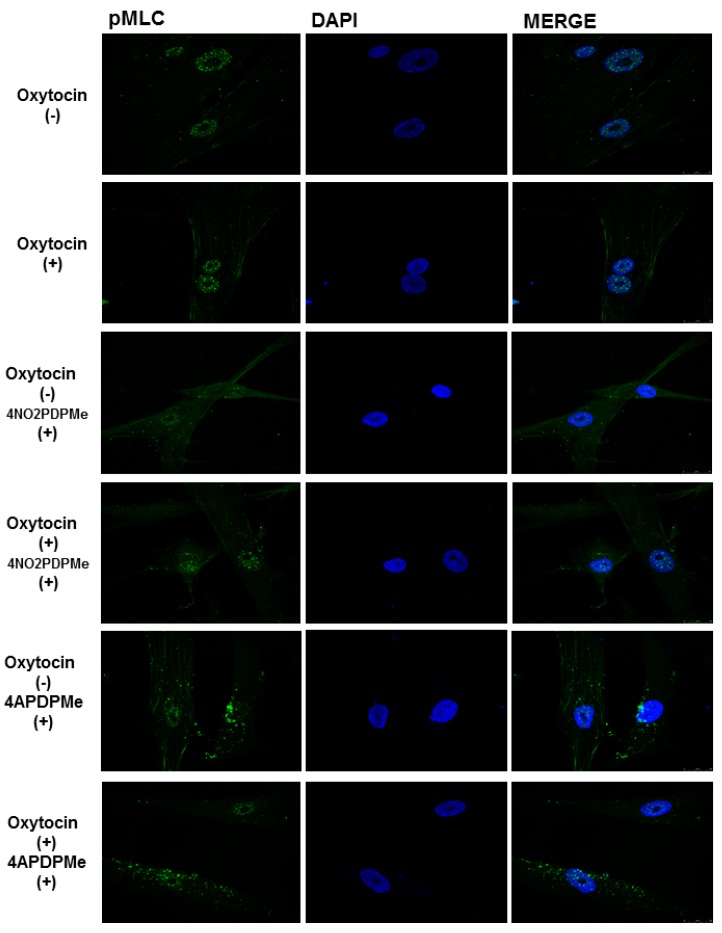
The effects of thalidomide analogs on OT-induced pMLC production in UtSMC cells. Basal pMLC production was faintly increased by OT addition (10 mIU/mL); this rise was augmented in the cytoplasm/cytoskeleton. Previous exposure of the cells to thalidomide analogs (1 mM) caused changes in either basal or OT-induced pMLC subcellular localization. DAPI: 4′,6-diamidino-2-phenylindole was used to stain the nucleus (blue).

The addition of OT to UtSMCs caused a small increase in the degree of phosphorylation of nuclear and cytoplasmic MLC compared with the control cells (Figure 6). The rise in pMLC was cytoplasmic rather than perinuclear, but this effect was marginal despite the high OT concentration used in presence of 1.8 mM Ca^2+^. Previous exposure of the cells to thalidomide analogs did not cause any significant reduction in OT-induced pMLC, even at 1 mM, which was the concentration that afforded the highest cAMP accumulation. Nevertheless, the presence of 4NO2PDPMe caused a change in pMLC localization by decreasing its normal intense nuclear localization while augmenting its presence in the cytoplasm; however, this effect was reversed by concomitant OT stimulation because the nuclear intensity of pMLC was maintained without a significant increase in either subcellular compartment. Likewise, 4APDPMe promoted a slight increase in the cytoplasmic pMLC level, although the addition of OT did not cause any significant reversion of the level or localization of pMLC.

## 3. Discussion

Since 1968, it has been reported that cAMP PDE is mainly present in the cytosolic fraction obtained from rat myometrium [32]. In 1978, relaxation of the pregnant human uterus induced by increased cAMP levels was found to result from the inhibition of human myometrial cAMP PDE [33]. The inhibition of PDE-4, mainly PDE-4B2, by selective agents, offers an important potential opportunity to modulate the myometrial activity of women in preterm labor with fewer side effects [31,34]; in fact, some PDE-4 inhibitors have been used alone or together with other cAMP-elevating drugs, such as β-mimetics, to improve the therapeutic outcome [35,36,37,38]. PDE-4 inhibitors are not only uterus-relaxant agents; they have also been shown to function as anti-inflammatory drugs in uterine tissues, which is important because inflammation provokes spontaneous uterine contractions [14,39,40]. Interestingly, all of the results obtained in the in vitro functional study of rolipram and thalidomide analogs as inhibitors of uterine contractions resembled those intended to evaluate the relaxant effect of diverse PDE-4 inhibitors on the spontaneous contraction of pregnant human myometrium [34,35,41], on tonic KCl-induced contractions in pregnant rat myometrium [42], and on in vivo spontaneous contractions in pregnant rats [38].

The relaxant effect of thalidomide analogs on spontaneous contractions was compared with rolipram. The range of rolipram concentrations used was not as wide as that reported by other authors, where a sigmoid concentration-response fits a model of interaction of two-stages in both non-pregnant and pregnant myometrium. In the present study, the IC_50_ of rolipram was in the micromolar range, whereas other authors have reported lower values [34,35]. Regarding the two-stage interaction model, it has been stated that PDE-4 isoforms can adopt two active conformers that are sensitive to rolipram, one-high affinity conformational state in the nanomolar range (HAPDE-4) and one low-affinity conformational state in the micromolar range (LAPDE-4). Both the correlation of HAPDE-4 with the side effects of rolipram and the correlation of LAPDE-4 with its therapeutic effects have been reported; in addition, the data indicate that the PDE-4 involved in the contractile process is in a LAPDE-4 conformational state near term [34]. This finding suggests that the IC_50_ of thalidomide analogs in the micromolar range may also result from their interaction with the LAPDE-4 conformer, and they might have fewer side effects than rolipram.

Spontaneous contraction was much more sensitive to inhibition than tonic contraction, an important difference that may be related to the detailed mechanisms controlling both types of contractions. Force generation in smooth muscle is driven by the phosphorylation of MLC, which is regulated by the equilibrium between the activities of MLCK and MLC phosphatase (MLCP). MLCK is activated by Ca^2+^-calmodulin, whereas MLCP is inhibited by the phosphorylation of its myosin-binding subunit (MLCPT1, at Thr853, which preserves contraction) by Ca^2+^-independent mechanisms, which precede an increase in MLC phosphorylation and in the force induced by a certain [Ca^2+^]_i_, a phenomenon known as “calcium-sensitization” [43,44]. Phosphorylation of MLCPT1 is achieved by several protein kinases, including Rho-associated kinase (ROCK) activated by the small G protein RhoA [43,45,46], which, in turn, is positively regulated by Rho-guanine nucleotide exchange factors (RhoGEFs) but negatively regulated by Rho-guanine nucleotide dissociation inhibitors (RhoGDIs) [47,48]. Several experiments conducted in vascular smooth muscle have demonstrated that ligand binding to metabotropic receptors (often G protein-coupled receptors) are capable of producing greater increases in force than KCl for a given [Ca^2+^]_i_; indeed, contractions induced by KCl are completely driven by membrane depolarization and Ca^2+^ entry through voltage-operated Ca^2+^ channels [8,26,27,28]. Therefore, although myometrial phasic contractions may be stronger than tonic contractions, they seem to be more sensitive to fine control; in fact, substantial differences have been found between uterine phasic and vascular tonic contractions due to the regulation of ROCK-induced phosphorylation of MLCP and MLC [8,43,46]. Furthermore, several crucial regulatory proteins involved in contraction are susceptible to phosphorylation by PKAs activated by elevated levels of cAMP; for example, ROCK1 and ROCK2 possess 45 and 43 sites, respectively, which are capable of being phosphorylated, some of which might inhibit their activity [47]. Moreover, the PKA-mediated inhibition of RhoA via the phosphorylation of Ser-188, which promotes its inhibitory association with RhoGDIs, has been reported [48,49]. In addition, many authors have proposed numerous other key proteins as the target of PKA–induced phosphorylation to inhibit contraction; for example, inhibition of phospholipase C activation, inhibition of Ca^2+^ entry through Ca^2+^ channels, activation of Ca^2+^ pumps and activation of large conductance Ca^2+^–activated K^+^ channels leading to membrane hyperpolarization, among others [11,50].

**Figure 7:**
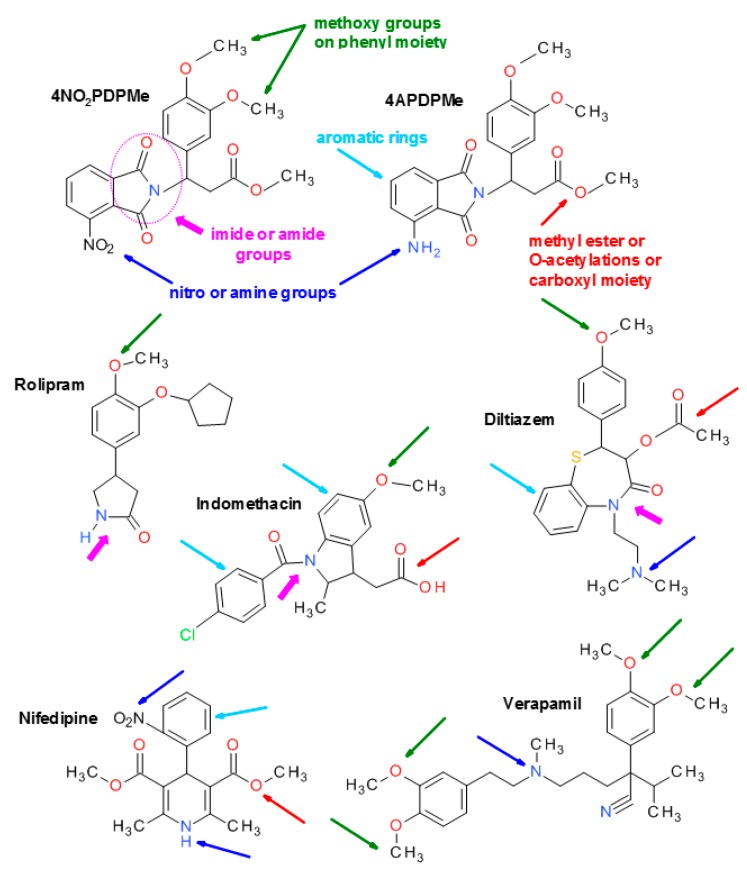
Chemical structures of thalidomide analogs and rolipram in comparison to some known Ca^2+^-channel blockers. All of them displayed a variety of similar moieties such as a dialkoxyphenyl group (methoxy or other *O*-alkyl substituents), nitro, imide and amide groups, a carboxyl moiety or its derived methyl ester as well as *O*-acetylation. Similar moieties are indicated by arrows and/or dashed lines in different colors.

The blockade of calcium entry by rolipram, 4NO2PDPMe or 4APDPMe emerged as a novel mechanism of action. Tonic contraction depends completely on membrane depolarization and Ca^2+^ entry through voltage-operated Ca^2+^ channels. However, cAMP-dependent relaxation appears to be mediated by a decrease in either intracellular [Ca^2+^]_i_ and/or MLC phosphorylation; nevertheless, cAMP concentrations can lead to the relaxation of tonic smooth muscle by mechanisms that are independent of changes in [Ca^2+^]_i_ or in the state of myosin light chain phosphorylation [51]. Because extracellular Ca^2+^ influx is essential for contraction, the present findings demonstrated that rolipram and thalidomide analogs inhibited the Ca^2+^–induced contractile response rather than the release of Ca^2+^ from storage sites, which occurs in the pregnant rat myometrium [52]. This interesting effect may be related to the chemical structure of these compounds because diverse chemical moieties of rolipram, 4NO2PDPMe or 4APDPMe are very similar to various very well-known specific Ca^2+^–channel blockers, such as nifedipine, verapamil, diltiazem and indomethacin, which are structurally similar to diverse PDE-4 inhibitors [16] and to different chemical families of Ca^2+^-channel blockers that relax smooth muscle regardless of the contraction stimulant [26] (see Figure 7). Indeed, novel 1,4-dihydropyridine-based (similar to nifedipine and nicardipine molecules) PDE-4 inhibitors, which share many functional groups, have been described [53]; moreover, the number of reports disclosing the effects of PDE-4B inhibitors on Ca^2+^–channel blockage has recently increased [54]. Because thalidomide analogs are much less potent than specific Ca^2+^–channel blockers, they may not pose a risk of cardiovascular side effects [1,3,4].

The Ca^2+^ entry blocking activity of the analogs coincided with their respective order of potency as relaxant agents in functional models, with their potency as TNF-α inhibitors, and with their PDA-4 inhibitory potency [19,20,21]. In this study, the concentration–cAMP amount curves for the thalidomide analogs in HeLa cells were similar to the cAMP levels induced by other PDE-4 inhibitors [31,38,42] or a β-mimetic [36] in myometrial tissue. However, 4NO2PDPMe was a more potent PDE-4 inhibitor than 4APDPMe, which may be because the potency values determined in functional studies of the human pregnant myometrium do not always parallel the potency values determined in biochemical studies for selective PDEs, and such differences may be related to the cell type and methodology applied [35], as well as to solubility and chemical stability [19]. Importantly, these thalidomide analogs belong to a family of immunomodulatory compounds that are potent PDE-4 inhibitors, but not teratogens, because all of them lack the glutarimide ring and thus fail to bind to cereblon, the primary target of the teratogenic action of thalidomide [20,22,23,55,56].

**Figure 8:**
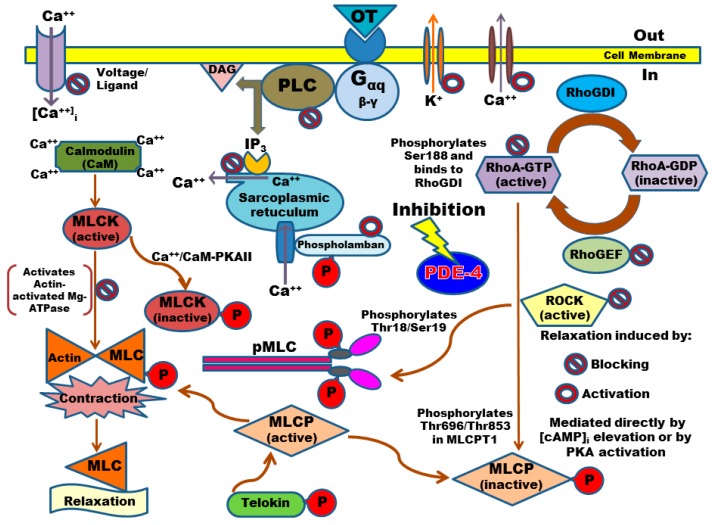
Schematic summarizing the possible protein phosphorylation targets of cAMP-activated PKA to induce relaxation in uterine smooth muscle as the mechanism of action of PDE-4 inhibitors; phosphorylation may activate or block protein targets. cAMP: adenosine 3′,5′-cyclic monophosphate; DAG: diacylglycerol; Gαq: protein G with subunit αq; IP_3_: inositol trisphosphate; MLC: myosin light-chain; MLCK: myosin light-chain kinase; MLCP: myosin light-chain phosphatase; MLCPT1: myosin-binding subunit; OT: oxytocin; P: phosphate group addition; PDE-4: phosphodiesterase-4; PKA: cAMP-dependent protein kinase; PLC: phospholipase-C; pMLC: phosphorylated myosin light-chain; RhoA: small G protein RhoA bound to guanosine triphosphate (GTP) or diphosphate (GDP); RhoGDIs: Rho-guanine nucleotide dissociation inhibitors; RhoGEFs: Rho-guanine nucleotide exchange factors; ROCK: Rho-associated kinase.

Nuclear, cytosolic, and cytoskeletal localization of PDE-4B2 and pMLC were corroborated in the human UtSMCs as the main pharmacological target [12,13] and as the pivotal mediator of contraction [57,58], respectively. The phosphorylation of MLC at Thr18/Ser19 in human uterine smooth muscle cells is a well characterized event that permits myosin-actin cross-bridging and is the hallmark biochemical event leading to contraction [59]. Hence, taking into account that UtSMCs have different behaviors than pregnant myometrial cells [60], OT induced a small, but evident, increase in pMLC [59]. Conversely, the analogs did not significantly diminish the increase in pMLC; this negative outcome may be explained by the common relaxation mechanisms shared by myometrial cells and tonic smooth muscles, in which increases in [cAMP]_i_ can lead to relaxation independently of changes in [Ca^2+^]_i_ or in the state of MLC phosphorylation [51]. However, the analogs provoked interesting changes in pMLC localization, considering that non-pregnant myometrial cells express more MLC than pregnant myometrial cells [61]. Changes in perinuclear or cytosolic/cytoskeletal pMLC localization suggest a cascade of events promoted by the differential subcellular compartmentalization of the increased levels of cAMP through the inhibition of PDE-4 by 4NO2PDPMe or 4APDPMe [57]. Indeed, PDE-4 inhibitors have been observed to increase the diffusion of cAMP from the site of synthesis; thus, PDEs may control local cAMP levels, the access of cAMP to the regulatory subunit of PKA and thus the state of activation of PKAs, which, in turn, may phosphorylate diverse target proteins, including MLCK, to produce pMLC [62]. Paradoxically, a similar route has been described for β1-adrenergic receptor antagonists; they locally increase cAMP levels without stimulating its direct production and thus augment PKA activity [63]. Finally, Figure 8 summarizes the possible protein phosphorylation targets of PKA to induce relaxation in uterine smooth muscle as the mechanism of action of PDE-4 inhibitors.

## 4. Materials and Methods

### 4.1. Thalidomide Analogs

The two thalidomide analogs methyl 3-(4-nitrophthalimido)-3-(3,4-dimethoxyphenyl)-propanoate (4NO2PDPMe) and methyl 3-(4-aminophthalimido)-3-(3,4-dimethoxyphenyl)-propanoate (4APDPMe) were synthesized following the synthetic route previously described [20,21]. The reaction conditions were modified and developed in our laboratory based on reported methods, and both analogs have been previously characterized using physicochemical and spectroscopic techniques [64,65,66,67]. Briefly, 4NO2PDPMe was prepared by condensation of 3-nitrophthalic anhydride with methyl-3-amino-3-(3’,4’-dimethoxyphenyl)propionate hydrochloride in refluxing acetic acid. 4APDPMe was prepared by the hydrogenation of 4NO2PDPMe catalyzed by 10% Pd/C in acetone.

### 4.2. Chemicals and Solutions

For all reagents, those with the best commercially available quality were acquired. The specific PDE-4 inhibitor rolipram, the nonselective PDE inhibitor IBMX, and the adenylyl cyclase activator forskolin were purchased from Sigma-Aldrich (St. Louis, MO, USA). All tested compounds were dissolved in dimethyl sulfoxide (DMSO) plus ethanol (1:2), and the maximum final concentration of DMSO was 0.33% in a total volume of 3 mL in each chamber in the functional studies or less in the remaining in vitro experiments. DMSO did not have any effect on the myometrial activity or the cellular viability. Ringer physiological salt solution was prepared as follows (mM): NaCl 144, NaHCO_3_ 30, KCl 6.2, KH_2_PO_4_ 1, MgSO_4_ 0.5, CaCl_2_ 2.5, glucose 11.1, pH 7.4.

### 4.3. Tissue Collection

Myometrial samples were collected from the mid-upper margin of the uterine incision of normal singleton term pregnant women (37–42 weeks of gestation) undergoing elective cesarean section. All cesarean sections were performed before the onset of labor for breech presentation, previous cesarean section, or maternal request. The age range of the women was 18–30 years old, and the average age was 23.2 years; none of the women suffered from any chronic disease. Written informed consent was obtained from each donor. The study was approved by the General Hospital Ethics Committee (Pachuca, Hgo., Mexico) and was performed in accordance with The Code of Ethics of the World Medical Association (Declaration of Helsinki).

### 4.4. In Vitro Functional Studies with Human Myometrial Strips

#### 4.4.1. Inhibitory Effect of 4APDPMe, 4NO2PDPMe, and Rolipram on Spontaneous Contraction

Biopsies were immediately placed in Ringer solution and transported on ice to the laboratory. The tissues were stored at 4 °C and used within 24 h of collection. Myometrial strips (8–10 mm in length by 3–4 mm in cross-section) were vertically mounted in isolated organ chambers containing 3 mL of Ringer solution for isometric tension recording using an isometric FT03C force transducer. Ringer solution in the organ bath was maintained at 37 °C and continuously bubbled with 5% CO_2_ in O_2_ (pH 7.4). The strips were equilibrated at a passive resting tension of 2 g throughout the experiments. Isometric tension was recorded using a polygraph model RPS-312 RM (Grass Telefactor, Boston, MA, USA). Data were acquired and analyzed using PolyVIEW software version 2.5 (chart rate of 2.5 mm/min in all records). The specimens were equilibrated for 2 h until spontaneous contractions were stable, and the bath solution was changed every 20 min. Two hours was considered sufficient time to observe the minimum required tissue responsiveness, which was defined as the stable frequency of at least two contractions/7 min, along with the lowest acceptable amplitude of 1 g (plus the resting tension of 2 g); tissues with poor spontaneous contractile activity were discarded.

Myometrial contractions (integral activity) were measured by the area under the curve (AUC) defined by the graphic isometric record over a 30-min period after stabilization. The inhibitory effects of 4APDPMe, 4NO2PDPMe and rolipram on spontaneous uterine contraction were expressed as follows:

% Inhibition = 100% − (AUCr/AUCi) × 100

where AUCr is the remaining AUC after uterine tissue exposition to drug, and AUCi is the AUC of the integral activity prior to any compound addition. A period of 30 min, before and after exposition to drugs, was considered sufficient to obtain stable and representative biological activity. Non-cumulative concentration–response curves were then generated for 4APDPMe, 4NO2PDPMe and rolipram for comparison, with the following range of concentrations: 32, 56, 100, 180, 230, 320 and 560 µM.

#### 4.4.2. Inhibitory Effect of 4APDPMe, 4NO2PDPMe, and Rolipram on Tonic Contraction Induced by High Levels of K^+^

To assess the effect of drugs on the tonic contractile response, strips of myometrium were contracted with a depolarizing solution (40 mM KCl) prepared by equimolar substitution of NaCl. Tissues that displayed two similar consecutive contractile responses were included in the study and recorded isometrically as described above. Each concentration of compounds was added upon tonic contraction 30 min after the stimulus (plateau phase) and maintained for an additional 30 min. The viability of the tissues was verified by adding a depolarizing solution to the strips of myometrium at the end of each experiment, revealing amplitude contractions similar to those at the beginning of the assay. Data were expressed as the percentage of the maximal tonic response induced by 40 mM KCl (100%). The test was repeated at least six times for each concentration to obtain the mean value and standard deviation; additionally, one control tissue was maintained in each experiment, which showed no variation in response to KCl-induced tonic contractions.

Data for the effect of thalidomide analogs or rolipram on both spontaneous and tonic contractions underwent a concentration-response curve analysis, which was performed using Sigma Plot version 10 software (Systat Software Inc., San Jose, CA, USA) to obtain the inhibitory concentration-50 (IC_50_) values, a drug concentration that produces 50% of the maximum inhibitory effect. The Emax is the maximum inhibitory response that can be produced by the highest concentration of the tested compound.

#### 4.4.3. Calcium Entry Blockade by 4APDPMe, 4NO2PDPMe, and Rolipram

To evaluate the possible blocking effect on Ca^2+^ entry by thalidomide analogs and rolipram, experiments were conducted in a calcium–free depolarizing solution. Myometrial strips were first placed in a standard depolarizing Ringer solution to obtain complete contraction provoked by 40 mM KCl, which was considered 100% amplitude, and the solution was then replaced with a Ca^2+^–free depolarizing Ringer solution modified with Ca^2+^ chelating 0.1 mM EGTA for 40 min. The different CaCl_2_ concentrations were then added in a cumulative manner (0.5–5 mM) to construct a control Ca^2+^–induced contractile response curve. The contractile response was expressed as the percentage yielded with respect to complete myometrial contraction. Similar experiments were repeated in the presence of the IC_50_ of thalidomide analogs or rolipram to assess tonic contractions over a 20 min period, prior to CaCl_2_ addition, and the results were compared with the control curve [28].

### 4.5. Measurement of cAMP Accumulation in Intact Cells

HeLa cells were routinely grown in DMEM supplemented with 10% fetal calf serum and 0.01% (*w*/*v*) penicillin/streptomycin in a humidified 5% CO_2_-enriched atmosphere at 37 °C. The cells were seeded in 24–well plates at a density of 250,000 cells/well. The day of the experiment, the cells were incubated (37 °C) in 250 μL Krebs–Ringer–HEPES buffer composed of the following (mM): NaCl 113, NaHCO_3_ 25, KCl 3, MgCl_2_ 1, KH_2_PO_4_ 1, CaCl_2_ 1.8, d-glucose 11, HEPES 20; the pH was adjusted to 7.4 with NaOH. In some wells, the non-selective phosphodiesterase inhibitor IBMX (1 mM) was used as a positive control for cAMP accumulation. This concentration was intended to completely inhibit phosphodiesterase based on the IC_50_ value of 7.5 μM reported for the inhibition of soluble, calcium-dependent phosphodiesterase activity in extracts from rat cerebral cortex; additionally, previous experiments have been performed to verify complete inhibition in the presence of 1 mM IBMX [68]. After 15 min, forskolin was added in a 10 μL volume and incubated for an additional 15 min. A final forskolin concentration of 3 μM was used to test the different concentrations of 4APDPMe or 4NO2PDPMe (10, 30, 100, 300 and 1000 μM) and, where required, the thalidomide analogs were added 30 min before forskolin. Time-course experiments were performed to establish the time that yielded the highest accumulation of cAMP induced by thalidomide analogs, and a 30 min period was selected.

Incubations were terminated with 25 μL ice-cold 1 M HCl. After neutralization with 25 μL of 1 M NaOH and 100 μL of 1 M Tris-HCl (pH 7.4), endogenous cAMP levels were determined using a competition assay, in which 50-μL samples were incubated in 125 μL of incubation buffer (in mM: 50 Tris-HCl, 100 NaCl, 5 EDTA, 5 mg/mL BSA, pH 7.0 at 4 °C) containing the PKA regulatory subunit (0.5 UI per sample) and [3H]-cAMP (10 nM). After 2.5 h at 4 °C, the reactions were terminated by filtration over GF/B filters pre-soaked in 0.3% polyethylenimine, followed by three washes with 1 mL of ice-cold deionized water. The retained radioactivity was measured by liquid scintillation, and the amount of endogenous cAMP present in each sample was calculated using a standard cAMP curve (10^−12^–10^−6^ M) [68]. Data for the effect of thalidomide analogs on the accumulation of cAMP in HeLa cells was subjected to a concentration-response curve analysis, which was performed using Prism version 5 software (GraphPad Inc., San Diego, CA, USA) to obtain the values of the effective concentration-50 (EC_50_), which was considered the concentration of drug that produces 50% of the effect between baseline and the maximum response.

### 4.6. Immunocytochemistry

Normal human uterine myometrial cells (UtSMCs) were purchased from Clonetics (San Diego, CA, USA) and grown in medium containing supplements and fetal bovine serum from a SmGM-2 Bullet Kit (Cambrex, Walkersville, MD, USA). UtSMC cells were trypsinized and seeded onto glass coverslips 2 days prior to use to achieve a confluence of 80% at the time of experimentation. The cells were maintained in a humidified 37 °C incubator with 95% air and 5% CO_2_.

To assess the level of the resultant pMLC, UtSMCs plated on coverslips were incubated for 30 min in the absence and presence of 1 mM 4NO2FDPMe or 4AFDPMe (the concentration that induced the maximal accumulation of cAMP in the previous experiment). Later, depending on the experiment, the cells were or were not exposed to oxytocin (OT, 10 mIU/mL) to provoke the phosphorylation of MLC because OT is the most potent biological inducer for this cell type [59]. After treatment with the compounds, the cells were washed three times in phosphate-buffered saline (PBS) and fixed with 4% formaldehyde for 10 min at room temperature. Subsequently, they were permeabilized for 2 min in PBS containing 0.2% Triton X-100, blocked for 20 min with 1% gelatin (*w*/*v*) and 1.5% fetal bovine serum (FBS) in PBS and incubated overnight at 4 °C with p-MLC (diphospho Thr18/Ser19, rabbit PAb) and PDE4B2 (T-19, goat IgG) antibodies (Santa Cruz Biotechnology, Santa Cruz, CA, USA). The final concentration of both antibodies was 1:25. The cells were then washed with PBS and incubated for 1 h at 4 °C with fluorescein-conjugated donkey anti-goat IgG (Santa Cruz Biotechnology). 4′,6-diamidino-2-phenylindole (DAPI) was used to stain nuclei (blue). The cell preparations were washed again with PBS and mounted on microscope slides with VectaShield (Vector Laboratories Inc., Burlingame, CA, USA); the samples were visualized on a confocal laser scanning microscope (TCP-SP5, Leica Microsystems, Heidelberg, Germany) using a Plan Neo Fluor 63× (NA = 1.4) oil-immersion objective.

### 4.7. Statistical Analysis

The data are expressed as the means ± SEM from determinations for each concentration (*n* = 6). The differences in IC_50_ (potency) and Emax (efficacy) among the compounds were determined by one-way ANOVA followed by a post hoc Bonferroni test using Sigma Stat software version 3.1 (Systat Software Inc., San Jose, CA, USA). In all cases, *p* < 0.05 was considered statistically significant.

## 5. Conclusions

In this work, the thalidomide analogs 4NO2PDPMe and 4APDPMe showed inhibitory effects on the spontaneous and tonic contractions of pregnant human myometrium, likely due to their inhibitory effect on PDE-4 and to their novel mechanism as calcium-channel blockers. This is the first report to propose a Ca^2+^ entry blockade effect of rolipram and these analogs. Although further studies are required, the immunomodulatory, anti-inflammatory, and uterus-relaxant properties of these non-teratogenic thalidomide analogs position them as potentially safe and effective tocolytic agents in a field that urgently needs improved pharmacological treatments, as in cases of preterm labor.

## Figures and Tables

**Table 1:** Rolipram and thalidomide analog IC_50_ and Emax values for myometrial spontaneous and tonic contractions.

Contraction	Spontaneous		Tonic KCl-Induced	
Compounds	IC_50_ (µM)	Emax (%)	IC_50_ (µM)	Emax (%)
Rolipram	22.74 ± 2.32	94.13 ± 7.53	98.45 ± 8.86	97.10 ± 8.73
4APDPMe	62.17 ± 5.61 ^a^	88.16 ± 7.93	125 ± 13.72	88.65 ± 7.09
4NO2PDPMe	128.30 ± 14.56 ^a,b^	67.99 ± 8.15	203.45 ± 18.31 ^a,b^	70.61 ± 6.78

Results are the mean value ± SEM from different concentration-effect curve analysis experiments (*n* = 6). IC_50_ = inhibitory concentration-50. Emax = maximum inhibitory effect. a = Difference vs. rolipram, b = difference vs. 4APDPMe, *p* < 0.05.

**Table 2:** Comparative analysis of Ca^2+^-induced contractile responses in the presence of rolipram and thalidomide analogs.

Ca^2+^-Induced Contractile Response (%)				
[Ca^2+^] (mM)	1	2	3	5
Control	34.21 ± 3.53	68.14 ± 4.45	81.09 ± 5.15	93.03 ± 5.06
4NO2PDPMe	23.32 ± 2.32	50.15 ± 3.19 ^a^	61.37 ± 3.32 ^a^	70.29 ± 3.54 ^a^
4APDPMe	13.23 ± 2.16 ^a^	30.32 ± 3.32 ^a,b^	47.18 ± 3.50 ^a^	55.34 ± 3.59 ^a^
Rolipram	5.35 ± 2.51 ^a,b^	18.12 ± 3.32 ^a,b^	28.58 ± 3.35 ^a,b,c^	35.28 ± 3.54 ^a,b,c^

Results are the mean value ± SEM from different experiments (*n* = 6). The control Ca^2+^-induced contractile response was obtained in a calcium-free Ringer solution and expressed as the percentage of the complete myometrial contraction achieved in response to 40 mM KCl in standard depolarizing Ringer solution, which was considered 100% amplitude. The drug concentration is the respective IC_50_ concentration for tonic contraction. a = Difference vs. control, b = difference vs. 4NO2PDPMe, c = difference vs. 4APDPMe, *p* < 0.05.
